# Focus on Extracellular Vesicles: New Frontiers of Cell-to-Cell Communication in Cancer

**DOI:** 10.3390/ijms17020175

**Published:** 2016-02-06

**Authors:** Chiara Ciardiello, Lorenzo Cavallini, Cristiana Spinelli, Julie Yang, Mariana Reis-Sobreiro, Paola de Candia, Valentina Renè Minciacchi, Dolores Di Vizio

**Affiliations:** 1 Division of Cancer Biology and Therapeutics, Departments of Surgery, Biomedical Sciences and Pathology and Laboratory Medicine, Samuel Oschin Comprehensive Cancer Institute, Cedars-Sinai Medical Center, Los Angeles, CA 90048, USA; chiara.ciardiello@hotmail.it (C.C.); lorecava@hotmail.it (L.C.); cristiana.spinelli@cshs.org (C.S.); julie.yang@cshs.org (J.Y.); mariana.sobreiro@cshs.org (M.R.-S.); valentina.minciacchi@cshs.org (V.R.M.); 2 Experimental Pharmacology Unit, Department of Research, IRCCS-Istituto Nazionale Tumori Fondazione G. Pascale, 80131 Naples, Italy; 3 Department of Experimental and Clinical Biomedical Science, University of Florence, 50121 Florence, Italy; 4 Istituto Nazionale Genetica Molecolare “Romeo ed Enrica Invernizzi”, 20122 Milan, Italy; paoladecandia@yahoo.com; 5 The Urological Diseases Research Center, Boston Children’s Hospital, Boston, MA 02115, USA; 6 Department of Surgery, Harvard Medical School, Boston, MA 02115, USA

**Keywords:** extracellular vesicles, exosomes, ectosomes, large oncosomes, microvesicles, cancer, intercellular communication, tumor microenvironment

## Abstract

Extracellular Vesicles (EVs) have received considerable attention in recent years, both as mediators of intercellular communication pathways that lead to tumor progression, and as potential sources for discovery of novel cancer biomarkers. For many years, research on EVs has mainly investigated either the mechanism of biogenesis and cargo selection and incorporation, or the methods of EV isolation from available body fluids for biomarker discovery. Recent studies have highlighted the existence of different populations of cancer-derived EVs, with distinct molecular cargo, thus pointing to the possibility that the various EV populations might play diverse roles in cancer and that this does not happen randomly. However, data attributing cancer specific intercellular functions to given populations of EVs are still limited. A deeper functional, biochemical and molecular characterization of the various EV classes might identify more selective clinical markers, and significantly advance our knowledge of the pathogenesis and disease progression of many cancer types.

## 1. Introduction

Extracellular vesicles (EVs) are structures of variable size (from 30 nm to a few μm), surrounded by a lipid bilayer, which are released from any type of cell into the extracellular space and are detectable in body fluids. EVs can exert pleiotropic biological functions, and can influence the microenvironment via the horizontal transfer of bioactive molecules, including proteins, lipids, DNA, and RNA [1,2]. EVs have been implicated in several physiological and pathological processes, such as inflammation, immune disorders, neurological diseases, and cancer [3,4,5]. One of the first lines of evidence that tumor cells shed membrane-vesicles was provided in 1978, when Friend and colleagues described them as “rare, pleomorphic membrane-lined particles ranging broadly in size between 400 and 1200 Å”, in cell lines derived from patients with Hodgkin’s disease [6]. A year later an independent study identified plasma-derived vesicles released by murine leukemia cells [7]. At the beginning of the 1980s, the shedding of plasma membrane EVs from pig hepatocellular carcinoma and mouse breast carcinoma cells helped to demonstrate that tumor EVs are carriers of procoagulant activity and thus participate in cancer thrombogenicity by activating the clotting system [8]. However, only twenty years later it was formally proven that EVs are not artifacts and can affect tumor progression by promoting angiogenesis, tumor invasion, and immune escape [9,10]. Since then, the number of reports on cancer-derived EVs has surged, and it is now well established that EVs contain functional proteins, microRNA, DNA, and mutated transcripts with oncogenic properties. In particular, it has been proposed by several studies that EV DNA may serve as a biomarker for cancer detection [11,12] and targeted therapy [13]. In addition, this very active field of investigation recently culminated in the demonstration that EVs can condition the pre-metastatic niche *in vivo*, a report that unequivocally corroborates active participation of EVs in cancer lethality [14].

## 2. The Variegated World of Extracellular Vesicles

Tumor cells release several types of EVs, which differ in size, biogenesis, and molecular composition. Two main categories of EVs have been described: exosomes and ectosomes. In addition, recent data point to the existence of additional subpopulations of EVs, which may express quantitatively and/or qualitatively different types of molecular cargo [15,16,17,18]. Moreover, Nakano and colleagues have recently observed that different subtypes of glioblastoma may activate different pathways of EVs biogenesis, due to the activation of diverse intracellular signaling [19].

Establishing an “EV population fingerprint” is highly relevant for two main reasons. On one hand, the definition of specific biological roles associated with specific EV categories may significantly advance our knowledge about the pathogenesis and the progression of disease. On the other hand, a deeper functional, biochemical and molecular characterization of diverse populations of EVs might identify more selective clinical markers, finely defining specific steps of tumorigenesis and potential avenues for therapeutic intervention.

### 2.1. Exosomes

**Figure 1:**
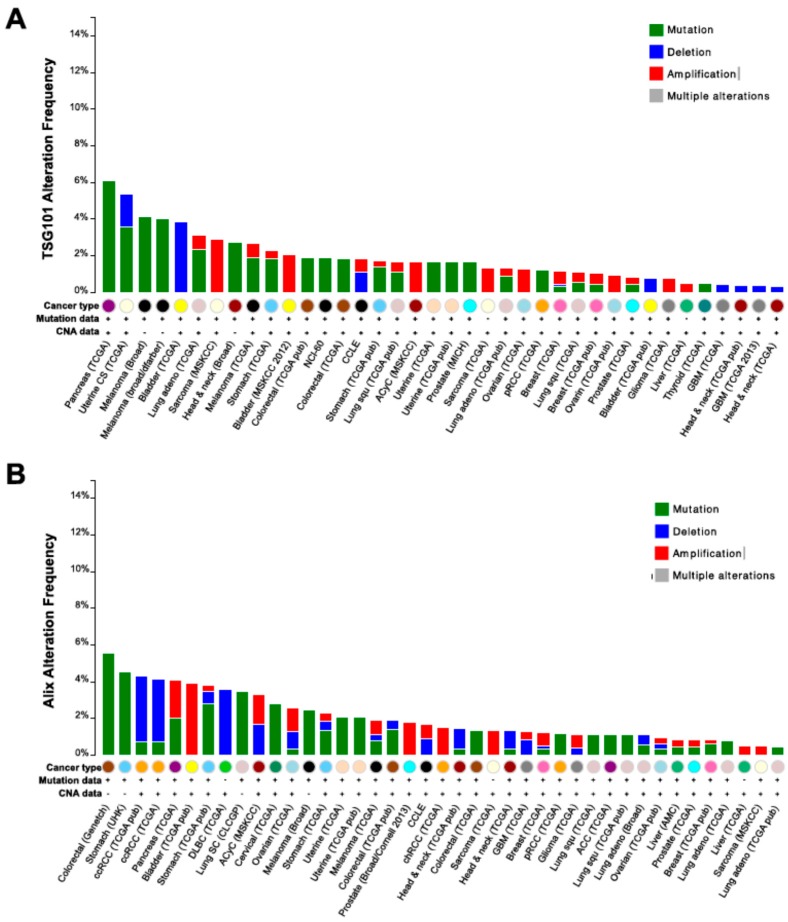
Genomic alterations of Tsg101 and ALIX in cancer. Frequency of copy number alterations and mutations of Tsg101 (**A**); and ALIX (**B**) across several tumor types. The results shown here are based upon data generated by the TCGA Research Network (Available online: http://cancergenome.nih.gov/).

*Exosomes* are nano-sized EVs (30–100 nm) that originate from the late endosomal trafficking machinery, are gathered intracellularly into multivesicular bodies (MVBs) and ultimately released as a result of MVB fusion with the plasma membrane [20]. An analysis of the proteins most frequently identified in exosomes and deposited in the online EV databases *ExoCarta*, *Vesiclepedia* and *EVPedia* [21,22,23,24,25] highlights the presence of the tetraspanin family members CD9, CD63 and CD81, the small actin-binding protein Cofilin1, heat shock proteins such as Hsp70 and Hsp90, and enzymes involved in cell metabolism, including Enolase1, Aldolase A, phosphoglycerate kinase 1 (PGK1) and lactate dehydrogenase A (LDHA) [26]. While most of these proteins have been shown to play a role in cancer progression, their identification in exosomes is not specific for cancer. Additional proteins that have more recently emerged as specifically associated with exosomes, and often absent in EVs other than exosomes, are Tsg101 and Programmed Cell Death-6 Interacting Protein (PDCD6IP), also known as ALG-2 Interacting Protein or, more commonly, as ALIX. Interestingly, these proteins are part of the ESCRT complex (ESCRT I and ESCRT III, respectively) that has been recently shown to play a direct role in exosomes biogenesis, with specific components differently affecting vesicle shedding [27]. While silencing of Tsg101 induces a decrease in exosomes production, the absence of ALIX leads to a specific increase in the release of larger EVs [27], suggesting a role for both proteins in the biogenesis of exosomes. However, conclusive results on the absence of Tsg101 and ALIX in non MVB-derived EVs are still lacking, neither is it clearly known whether they can be identified in exosomes from all cell systems, including cancer. Moreover, despite findings demonstrating that Tsg101 and ALIX can interact, Tsg101 seems to play a direct role in cancer [28], whereas the function of ALIX is generally associated with programmed cell death [29]. Interestingly, both genes are mostly mutated, albeit at extremely rare frequency, in human tumors, as demonstrated by data generated by the TCGA Research Network (Available online: http://cancergenome.nih.gov/) (Figure 1).

### 2.2. Ectosomes

*Ectosomes*, also reported as membrane particles, nanoparticles, matrix vesicles and microvesicles (MV), are cell surface-derived EVs typically larger than exosomes, originating from direct budding from the plasma membrane [30]. This category includes apoptotic bodies (ABs) and possibly large oncosomes (LO), which derive from apoptotic and non-apoptotic membrane blebbing processes, respectively [31,32,33]. Ectosome shedding can be induced by alterations in the asymmetric distribution of phospholipids in the plasma membrane, which results in the exposure of phosphatidylserine (PS) and phosphatidylethanolamine (PE) in the extracellular layer of the membrane. This information derives mostly from studies on MV [34,35] and the phenomenon of ectosome shedding is stimulated by increased levels of intracellular Ca^2+^ in different cell types including cancer [36]. Cancer-derived MV are enriched in the ADP-ribosylation factor 6 (ARF6), which functions as a promoter of EV shedding from prostate and breast cancer cell lines [37]. Importantly, interrogation of publicly available prostate cancer expression datasets demonstrates that ARF6 mRNA levels are higher in prostate cancer compared with benign tissue [38].

Cancer ectosomes, similarly to other EV populations, represent a valuable reservoir for molecules functionally involved in cancer progression and are representative of their cell of origin [37,39,40] hence they make appealing candidates for the identification of circulating biomarkers. Despite the similarities in the molecular composition of ectosomes and donor cells, it is becoming evident that the internal composition of ectosomes, as well as the composition of the ectosome membrane, are not a mere reflection of the cytosol and cell membrane of the cell of origin [37,39]. Unraveling the mechanisms of molecular sorting in ectosomes could shed light on “intercellular messaging” that might occur at high rates in cancer.

### 2.3. Apoptotic Bodies

*Apoptotic bodies* (*ABs*) are particles of relatively large size (1–4 µm), released by tumor cells and other cell types upon the trigger of the cellular collapse that results in *karyorrhexis* (nuclear fragmentation), increase in membrane permeability, and externalization of phosphatidylserine (PS) [41,42]. Apoptotic membrane blebbing is a well-studied phenomenon that occurs during the late stages of programmed cell death, and is the result of caspase-mediated cleavage and consequent activation of ROCK1 [43]. It has been reported that ABs contain nuclear material, which might be functional. However, the results suggesting that ABs, through the horizontal transfer of oncogenes from cancer to recipient cells, participate in cancer development [44] needs further investigation *in vitro* and possibly *in vivo* [45]. Importantly, although an exchange of cancer-derived DNA has been reported in prostate cancer cells [46], whether this mechanism has functional consequences is still unknown. Another role suggested for ABs is that they can act by “dispatching suicide notes” to the surrounding environment. In fact, in early phases of apoptosis, AB membranes display increased permeabilization, allowing them to release proteins into the microenvironment. This, in turn, prepares the surrounding cells for the catastrophic loss of membrane integrity that affects apoptotic cells during secondary necrosis [47].

### 2.4. Large Oncosomes

*Large Oncosomes* (LO) represent an additional class of tumor-derived EVs, so called because of an atypically large size and abundant oncogenic cargo [31,38]. Similarly to ectosomes, this EV population might originate directly from plasma membrane budding and, like MV, these particles express ARF6 [38]. LO formation is particularly evident in highly migratory, aggressive tumor cells with an amoeboid phenotype [31,38], and experiments in different cell lines indicate that LO can form as bioproducts of non-apoptotic membrane blebs used by amoeboid cells as propulsive forces to migrate [32]. Quantitative analyses of LO has shown that even if LO diameter can vary from 1 to 10 µm and larger, the mode of distribution of LO diameters, calculated on hundreds of cells, is 3–4 µm, and some of them can reach several µm in size, considerably larger than any type of EV described so far. LO have been identified in several cancer systems, and comparative experiments between tumor and benign cells indicate that they are specifically released by tumor cells, whereas their detection in benign cell systems is negligible. Consistently, LO-like features have been described in human prostate cancer sections *in situ*, but not detected in benign tissue [38]. LO shedding is common to several tumor types, including prostate, breast, bladder, lung cancer, and other tumors ([38] and unpublished observations) and is enhanced by silencing of the gene encoding the cytoskeletal regulator Diaphanous related formin-3 (DIAPH3), or by activation of the epidermal growth factor receptor (EGFR) and overexpression of a membrane-targeted, constitutively active form of Akt1. Importantly, LO have been identified in the circulation of mice and patients with metastatic prostate cancer, suggesting that these EVs are potentially useful sources of clinical biomarkers [48].

EVs in the size range and appearance of LO have been recently described with different names including: (1) giant vesicles, identified in ERα-positive breast cancer cells and tumor tissues [49]; (2) migrasomes, large round structures containing numerous vesicles (pomegranate-like structures), which depart from retraction fibers of migratory benign cells [50]; and (3) tumor-derived MV originating from amoeboid-like tumor cells, in which VAMP3 seems to regulate the delivery of MV cargo to regions of high plasma membrane blebbing. MV appear to be released through blebbing during migration [51]. Whether these three types of EVs are distinct from LO and use different mechanisms to play their extracellular functions is currently unknown.

Despite the effort to reach a consensus on vesicle nomenclature and classification, it is becoming evident that the biochemical composition and the biological function of different EV populations derived from the same cellular system overlap, at least in part [52]. Furthermore, current methods of isolation do not discriminate between exosomes and ectosomes because physical properties that can unambiguously distinguish between the two EV types have not been fully characterized and specific molecular markers are still lacking [37,53,54]. However, despite the current limitations, a recent study indicates that RNA profiles of exosomes, ectosomes, and ABs differ [55]. Therefore, regardless of the confusing and frequently inappropriate terminology used to define particular EV populations, it is clear that tumor cells release a spectrum of EVs that might all functionally participate in the biology of the disease. A partial snapshot of the vesicle types described above is presented in Table 1. A more comprehensive introduction into EVs, particularly exosomes, is provided in a focus edition by Kalra *et al.* [56].

**Table 1:** Populations of Extracellular Vesicles.

Vesicle Type	Size	Origin	Pathway	Cargo	Ref.
Exosomes	30–300 nm	MVB fusion with the plasma membrane	Tsg101 and ALIX dependent	Tsg101, ALIX, CD9, CD63, CD81	[27,57]
Ectosomes	0.05–1 μm	Budding from the plasma membrane	ARF6, RhoA, PS exposure dependent	ARF6	[37,40]
Apoptotic Bodies	1–4 μm	Budding from the plasma membrane	Apoptosis-related pathway	Annexin V, Caspase 3	[42]
Large Oncosomes	1–10 μm	Budding from the plasma membrane	EGFR, Akt1, Cav-1 and DIAPH3-loss dependent	ARF6, CK18, GAPDH	[31,38,48]
Giant Vesicles	3–42 μm	Budding from the plasma membrane	17-β-estradiol dependent	Not Identified	[49]
Migrasomes	0.5–3 μm	Budding from retraction fibers	Integrin and migration dependent	TSPAN4	[50]

## 3. Cancer Extracellular Vesicles and the Tumor Microenvironment

**Figure 2:**
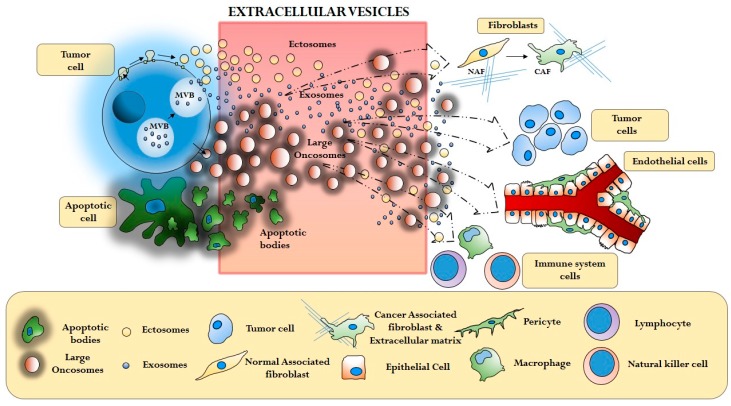
Extracellular Vesicle (EV)-mediated interaction between cancer cells and different components of the tumor microenviroment (Cancer Associated Fibroblasts—CAF, extracellular matrix—ECM, Tumor cells, Endothelial cells, Immune system cells).

The molecular mechanisms regulating the functional interaction between cancer cells and the microenvironment have been the subject of active investigation, and historically are considered to be mediated by small molecules, cytokines and growth factors. Today we know that cancer cells also communicate through EVs, thus transferring functional information from cell to cell at the paracrine level (Figure 2). Recent reports are leading to the conclusion that EV cargo influences the stroma by activating molecular pathways that differ, at least in part, from the ones modulated by soluble factors [58].

### 3.1. Cancer Cells and Cancer Associated Fibroblasts (CAFs)

Cancer associated fibroblasts (CAFs) are one of the most abundant cell type in the tumor microenvironment. Upon exposure to cancer cells, CAFs alter their phenotypes [59]. In turn, when co-cultured or exposed to conditioned media from CAFs, cancer cells demonstrate increased aggressiveness, which might depend on CAF-induced stemness and metabolic reprogramming [60,61,62,63]. Both cancer cells and fibroblasts can produce different species of EVs, which seem to actively participate in the mutual interplay between these cells, and this is crucial to tumor progression [58,64,65,66,67].

A recent study demonstrated that tumor-derived exosomes can modify the stroma to promote tumor growth by supporting angiogenesis and accelerating tumor cell proliferation [58]. Furthermore, the TGF-β associated with tumor exosomes can trigger fibroblast differentiation into a myofibroblast or CAF phenotype, with increased levels of α-smooth muscle actin (α-SMA). The uptake of tumor-derived exosomes by fibroblasts also results in the deposition of a hyaluronic acid pericellular coat leading to increased contractile activity [64]. Similarly, treatment with ovarian and breast cancer cell-derived exosomes induces, in adipose tissue derived stem cells, the typical characteristics of tumor-associated myofibroblasts [68,69]. Additionally, the Extracellular Matrix Metalloproteinase Inducer (EMMPRIN) [65] contained in exosomes and released by lung carcinoma cells is able to enhance the expressionof Matrix Metalloproteases (MMPs) in fibroblasts, with dramatic repercussions on tumor progression and metastasis.

Increasing attention has been placed to the role of cancer-derived ectosomes on the tumor microenvironment. Tumor ectosomes have been reported as carriers of cathepsins [70], MMPs, uPA and have been studied as promoters of the proteolytic cascade required by cancer cells to degrade the extracellular matrix and invade the surrounding environment [71]. Antonyak and colleagues demonstrated that ectosome-like MV, derived from different human cancer cells (breast carcinoma and glioma cells), can transform normal fibroblasts and enhance their survival abilities by transferring tissue transglutaminase (tTG), an enzyme that can cross-link Fibronectin (FN) on the ectosomes, enhancing the role of FN in potentiating activation of integrins and their downstream effectors, focal adhesion kinase (FAK) and ERK [72]. Additionally, LO can functionally influence the stroma, inducing Akt1 signaling pathway activation, expression of genes implicated in prostate cancer metastasis and functional changes in cancer-associated fibroblasts [38,73].

Evidence that exosomes derived from fibroblasts exert a conditioning activity on cancer cells is still very limited. One of the very first studies on the topic, showing that CAF-derived exosomes stimulate breast cancer cell motility, protrusive activity and metastasis, was published in 2012 [66]. The salient discovery of this seminal study is that CAF-derived exosomes can be taken up by breast cancer cells and then loaded with Wnt11 produced by the recipient cells. This results in CAF-derived exosomes, in which CD81 and Wnt11 colocalize in trans, which can activate planar cell polarity (PCP) signaling in an autocrine manner in cancer cells, thus promoting migration. A more recent report demonstrates that exosomes produced by dermal fibroblasts, in which the tissue inhibitor of MMPs (TIMP) gene family has been knocked down with consequent acquisition of a CAF phenotype, are enriched in Disintegrinand metalloproteinase domain-containing protein 10 (ADAM10), and can enhance breast cancer cell motility by activating RhoA and Notch signaling [67]. Notably, mass spectrometry analysis of fibroblast-derived exosomes indicates that these EVs stimulate migration and invasion of target cells and can also play a role in metabolic reprogramming and extracellular acidification [66,67]. All together, these findings indicate that EVs can be exchanged reciprocally between cancer cells and CAFs, and that most of the biological changes that occur in either compartment as a result of cancer progression, including stemness, invasion, extracellular acidification, and metabolism reprogramming are mediated by specialized EVs. However, comparative functional analysis of the functional influence exerted by different populations of EVs on the stroma are still almost completely missing.

### 3.2. Immune System Regulation by Cancer Extracellular Vesicles

The uptake of tumor derived EVs by immune cells seems to have functional consequences on the immune microenvironment, which can result in either escaping the immune response or in activating immune suppression. Whether different populations of EVs elicit distinct types of immune responses, and the molecular mechanisms underlying the transferring to and the modulation of the immune microenvironment by receptors, proteins, RNA, and DNA carried in EVs, are all still largely unknown. Nonetheless, studying how immune cells are educated by tumor-derived EVs, thus facilitating tumor cell escape strategies, is crucial in order to develop cancer vaccines and new cancer treatments.

One way tumor EVs educate the immune microenvironment is by transfer of information to monocytes. Baj-Krzywirzeka and colleagues [74] characterized a number of cell surface markers and mRNA transcripts in EVs from pancreatic adenocarcinoma (HPC-4), colorectal carcinoma (DeTa), and lung carcinoma (A549) cells and they found that IL8, VEGF, HGF, and CD44 mRNA were expressed at high levels in EVs, suggesting a function for these messengers in monocyte re-education. Moreover, the interaction of EV cell surface proteins with monocytes alters CCR6 and CD44v7/8 expression and activates Akt, resulting in increased chemo-taxis and cell survival. All these events may result in the recruitment of monocytes into the tumor tissue, where they can differentiate into tumor-associated macrophages and support tumor initiation, local progression, and distant metastasis [75].

Frequent targets of tumor-derived exosomes are CD8+ T lymphocytes. Wieckowski and colleagues [76] demonstrated that MV from head and neck squamous cell carcinoma (PCI-13) carry the cancer testis antigen MAGE 3/6, and FasL that, once transferred to CD8+ T cells, induce their cell death. Another study demonstrated that EVs can be collected from the sera of patients with acute myeloid leukemia (AML), which contains higher levels of membrane associated TGFβ-1 and FasL than control sera [77] and that the treatment of Natural Killer cells with AML patients’exosomes results in TGFβ-1-mediated cytotoxicity of the target cells. On the other hand, tumor exosomes have also the ability to induce immune suppression by activating CD4+/CD25+Foxp3+ regulatory T cells (*Treg*) via TGFβ-1.

The induction of an efficient adaptive immune response against the tumor requires dendritic cell cross-processing and presentation of tumor antigens to T cells. Rughetti and colleagues characterized a population of EVs containing the tumor associated MUC1, a glycoprotein that can bind the MHC groove and induce activation of CD8+ T cells, which can influence its processing, in dendritic cells. Importantly, dendritic cells can also internalize MUC1-positive EVs obtained from ascites of patients with stage III or IV ovarian cancer [78]. It is evident that, by eliminating the lymphocytes that have the ability to recognize and kill tumor cells, or by activating the regulatory suppressive branch of the adaptive response, cancer can escape immune attack and enhance its own survival [79].

### 3.3. Cancer Extracellular Vesicles and the Endothelium

The genesis of new blood vessels, known as angiogenesis, is an important step in cancer progression. Establishing the tumor vascular network is essential for cancer cell proliferation because tumor cells depend on blood for the supply of oxygen and nutrients and for the removal of waste products. The vascular supply becomes even more crucial to the cells during metastatic invasion, and facilitates the entrance of tumor cells in the bloodstream to colonize distant organs in the body [80,81].

Importantly, tumor exosomes contain key pro-angiogenic factors directly linked to endothelial cell migration and the induction of new blood vessel formation, such as Vascular Endothelial Growth Factor (VEGF), overexpressed in the majority of tumors [82,83], Epidermal Growth Factor like domains (EDIL-3/Del1) in bladder cancer cell-derived exosomes [84] and Annexin A2 (ANXA2), one of the most abundant proteins of glioblastoma cell-derived exosomes [85]. Kucharzewska and colleagues showed that exosomes derived from glioblastoma cells in hypoxic conditions are potent inducers of angiogenesis through phenotypic modulation of endothelial cells [86]. Furthermore, it has been shown that exosomes derived from renal cancer cells induce up-regulation of VEGF mRNA and protein levels in human umbilical vein endothelial cells (HUVECs), thus promoting angiogenesis [87].

Recent studies have demonstrated that tumor exosomes contribute to angiogenesis by transferring miRNAs [88]. Taverna and colleagues reported that exosomes derived from chronic myeloid leukemia are enriched with miR-126, which can be delivered to endothelial cells and down-regulate its molecular targets, CXCL12 and VCAM1. This results in enhanced migration of the cancer cells toward the endothelial cells in a co-culture model [89]. The release of exosomes enriched with miR-92a from leukemic cells induces down regulation of integrin α5 in endothelial cells and increases angiogenesis [90]. Additionally, exosomes derived from lung adenocarcinoma, enriched with miR-210, promote tube formation in HUVECs by down-regulating its target Ephrin A3, a known inhibitor of angiogenesis [91].

Recently, a functional role for large oncosomes (LO) in endothelial cell signaling and activities has been described: LO have been observed to induce migration of mouse dermal endothelial (MDEC) and tumor endothelial cells (TEC), suggesting that these EVs might disrupt the integrity of endothelial cell junctions and thereby increase blood vessel permeability *in vivo* [38]. However, additional studies to test whether the results on endothelial cell migration can be reproduced by using canonical angiogenesis assays *in vitro* are needed to fully understand the biological consequences of the cross-talk between LO and the endothelium. Further experiments are also necessary to determine if these atypically large EVs can be internalized into endothelial cells. Finally, although it has been demonstrated that LO extracted from the plasma of mice with prostate cancer can stimulate migration of endothelial cells, the capability of these EVs to cause endothelial leakage in animal models has yet to be tested. Additionally, the molecular mechanisms underscoring this and other biological functions of LO are still largely unknown [38].

While different EV populations have been studied and demonstrated to be involved in angiogenesis, whether they cooperate or not, and to what extent each population intervenes in specific phases of angiogenesis and metastasis is still a topic of investigation.

### 3.4. Extracellular Vesicles and the Transfer of Biological Information between Cancer Cells

EV-mediated intercellular communication is not limited to the interactions between tumor cells and the microenvironment described above, but rather occurs within the composite puzzle of heterogeneous cells that populate the tumor. Among the different roles EVs can play in extracellular communication in cancer, one fascinating hypothesis is that EVs might function by horizontal transfer of genes with pre-existent mutations. While the intercellular transfer of oncogenic DNA has often been attributed to the uptake of apoptotic bodies (ABs) [44], reports indicate that EV populations corresponding to exosome and MV subtypes can mediate single- and double-stranded DNA transfer respectively [92,93]. In particular, H-ras-transformed rat epithelial cells can shed EVs containing chromatin-associated double-stranded DNA fragments covering the entire host genome, which can transfer full-length H-ras to recipient cells, stimulating their proliferation and inducing phenotype changes [92]. Another emerging important function seems to be related to the transfer and propagation of drug resistance. Almost unavoidably, chemotherapy results in changes in the biology of cancer cells, which consequently leads to chemoresistance. The traditional view believes that cells with endogenously acquired chemoresistance will survive and propagate upon treatment. In addition, a new hypothesis suggests that chemoresistance can also be transmitted horizontally from one cell to another through the exchange of EVs. Indeed, Zhang and colleagues recently described EV-mediated transfer of resistance to Paclitaxel in ovarian cancer [94]. P-glycoprotein (P-gP), encoded by the multidrug resistance gene-1 (MDR-1), which is associated with resistance to a variety of anticancer agents, is delivered through EVs from resistant to sensitive cells that then acquire the resistant phenotype. Using a different mechanism, exosomes can indirectly modulate P-gP by transferring to recipient cells TrpC5, a Ca^2+^ permeable channel [95]. This leads to increased concentrations of intracellular Ca^2+^ and consequent increase of P-gP levels, and intercellular transfer of drug resistance. This seems to occur between different cell types, including cancer and endothelial cells [96]. Intriguingly, an additional novel molecular mechanism by which sensitive cells become chemoresistant via exosome-mediated horizontal communication is mediated by miRNA and other non-coding RNAs [97]. For example, miR-222, which is cargo of breast cancer exosomes can target PTEN, thus activating the PI3K pathway, stimulating cell proliferation and promoting chemoresistance to Adryamycin and Docetaxel in cells sensitive to these drugs. However, the specific mechanism by which the sensitive cells became resistant is not known [98]. EVs can also play a protective role in chemoresistance. This is the case for a study demonstrating that EVs can be directly involved in the sequestration, transport and expulsion of Cisplatin from cancer cells [99], suggesting that, by using a sophisticated disposal mechanism, cancer cells might escape drug treatments.

In the future, it would be useful to investigate the extent to which specific types of EVs are implicated in drug chemoresistance and if the transfer of entire genes can modify the genomic make up of the recipient cells thus modulating response to the drugs intrinsically, before initiating drug treatment [100]. Further information on various aspects of EVs is provided in the other reviews of this focus edition [56,101,102,103,104].

## 4. Extracellular Vesicle Associated Cargo with Particular Emphasis on MicroRNA

The molecular content of EVs seems to be highly dependent on the source and conditions of the system at the time of vesicle biogenesis. For example, the abundance of the RNA and protein content of endothelial cell-derived exosomes is altered after exposure of the endothelial cells to different types of stress, including hypoxia, treatment with TNFα, or exposure to high concentrations of glucose and mannose [105]. Moreover, the overexpression of the ErbB2/HER2 receptor tyrosine kinase can alter the proteome of two different populations of EVs derived from mammary luminal epithelial cell lines in comparison with parental cells, suggesting complementary roles for certain biological pathways, and possibly a different response to HER2 upregulation in different types of EVs [106]. A plethora of studies arose on proteomic analyses of EVs from different organ systems [107,108], as it has been discussed by recent reviews [109].

Particular attention has recently been paid to the study of microRNAs (miRNAs) as key regulatory molecules in several diseases, including cancer. In bodily fluids, miRNAs have been identified either in complex with the argonaute RISC catalytic factor AGO2 [110,111] or within EVs [39,112]. Kosaka and colleagues demonstrated that incorporation of miRNA into intraluminal vesicles of MVBs is controlled by neutral sphingomyelinase 2 (nSMase2), a regulator of ceramide biosynthesis [113]. Exosome shedding is potentiated by nSMase2, and its inhibition reduces the release of exosomes containing miRNA. This result has been confirmed in another study, demonstrating that RAB27A and RAB27B, two small GTPases that regulate secretory pathways, increase miRNA export from bladder carcinoma cells [114]. Vallarroya-Beltri and colleagues demonstrated that the miRNA sequence motif is important for miRNA loading into exosomes. This conclusion derives from mutagenesis experiments demonstrating that the heterogeneous nuclear ribonucleoprotein A2B1 (hnRNPA2B1) binds miRNAs directly through the recognition of their mature sequence, and controls their sorting in EVs. In exosomes, hnRNPA2B1 is sumoylated, and this post-translational modification facilitates its binding to miRNAs [115]. The EV-loading efficiency seems to depend not only on the RNA sequence but also on the cell type and physiological state. As a consequence, some small RNAs appear to be preferentially exported into EVs, while others are retained within the cell [116]. Whether these crucial mechanistic insights are applicable to cancer EVs remains to be proven.

Numerous studies have reported enrichment of miRNAs in EVs released by different types of cancer cells and present in biological fluids from cancer patients, resulting in the identification of promising markers of disease prognosis [117]. These EV miRNAs also play a functional role that is becoming increasingly recognized. A recent study suggests that decreased export of tumor suppressor miRNAs might be a mechanism of pro-metastatic function exerted by EVs *in vivo* [114]. One example is represented by miR-200, highly expressed in EVs from metastatic breast cancer cell lines, and responsible for enhanced metastatic abilities of less aggressive cells upon intercellular transfer [118].

Remarkably, Melo and colleagues have recently demonstrated the presence, in cancer but not in normal cell-derived exosomes, of AGO2 that, together with the RISC-Loading Complex (RLC), display cell-independent capacity to process pre-miRNAs in mature transcripts [119]. This study provides evidence that cancer exosomes, including those derived from breast cancer patient sera, can confer tumorigenic behavior to epithelial cells in a Dicer-dependent manner, and strongly reinforces the rationale for the development of exosome based therapies.

Surprisingly, the first quantitative and stoichiometric evaluation of exosome-derived miRNA demonstrates the presence of less than one miRNA copy per exosomes [120]. The authors propose two alternative models, a low-occupancy/low-miRNA concentration model, where a small number of exosomes carries a low concentration of miRNA, and a low-occupancy/high-miRNA concentration model, where rare exosomes carry many copies of a given miRNA. This study highlights the necessity to expand our current methodologies and approaches to more deeply investigate the functional role of EV miRNA cargo along with its potential use as a biomarker. It also corroborates the hypothesis that large EVs might contain higher amounts of miRNA [73]. Quantification of miRNA in EVs is an exciting topic in cancer research also because the amount of extracellular miRNA seems to be upregulated in the plasma of patients bearing tumors [121,122].

## 5. Conclusions

EVs are involved in several, if not all aspects, of tumor development and progression due to their apparently fundamental role in packaging and delivering molecular messages intercellularly. The potential implications, both diagnostic and therapeutic, of a deeper EV characterization, are widespread. Despite the current limitations, our abilities to specifically characterize EV populations are constantly improving. Furthermore, different EVs might all coexist at the same time within the tumor microenvironment and possibly cooperate or antagonize each other in the intercellular exchange of messages. In line with this concept, it might be helpful to functionally study different EV populations in a comparative manner or in combination. Finally, additional studies are necessary to clarify whether EV release is primarily a mechanism for spreading cancer-associated activities within the tumor microenvironment and in the circulation, if it is a mechanism of defense adopted by cancer cells to survive during disease progression, or both.
